# Using population register data and capture-recapture models to estimate over-coverage in Sweden

**DOI:** 10.1038/s41598-024-82547-9

**Published:** 2024-12-18

**Authors:** Bruno Santos, Eleonora Mussino, Sven Drefahl, Eleni Matechou

**Affiliations:** 1 CEAUL – Centro de Estatística e Aplicações, Faculdade de Ciências, Universidade de Lisboa, Lisbon, Portugal; 2 Stockholm University Demography Unit -SUDA, Stockholm University, Stockholm, Sweden; 3 University of Kent, School of Mathematics Statistics and Actuarial Science, Canterbury, UK

**Keywords:** Over-coverage, Capture-recapture models, Register data, Statistics, Applied mathematics

## Abstract

Over-coverage occurs when individuals who reside in a country leave or pass away, and this demographic event is not recorded in population registers, leading to population size overestimation. This problem can have important policy and decision-making consequences. With the increased reliance on incomplete but overlapping official registers for documenting whole populations or subgroups of populations, there is a need for more sophisticated modelling techniques that reliably estimate population size, and hence over-coverage, from such registers. Previous approaches have considered multiple systems estimation (MSE) for monitoring over-coverage, but MSE does not naturally extend to cases where individuals are followed over time. In this paper, motivated by the case study of Sweden, we develop a capture-recapture (CR) modelling framework for population registers that allows us to estimate the population size each year, the probability of presence for each individual in the population, conditional on their records, each year and to quantify the effect of demographic characteristics on the probability of emigration and re-immigration, amongst other parameters. Our results suggest that the CR approach, which accounts for the whole time series for each individual, gives a more realistic estimate of the population size compared to existing, deterministic approaches, especially when considering the subgroup of newly arrived individuals, and that it provides new insights on individual behaviour in terms of migration patterns than existing MSE approaches.

## Introduction

Policy and decision makers require correct information on population sizes of various groups and subgroups across different locations and periods. There is growing reliance in utilizing population register data, instead of census data, for accessing this information. Register data are routinely collected for administrative purposes and consequently they are often readily available and updated continuously. Registers are, however, not perfect, in that not all individuals in a given population are observed in at least one register. Multiple systems estimation (MSE)^1^ models have been successfully used in these cases, estimating the number of unobserved individuals and therefore total population sizes in problems related to human trafficking^2,3^, drug users^4^, and modern slavery^5^, among others.

A second common issue is the overestimation of population sizes, called over-coverage. Over-coverage occurs when individuals who reside in a country leave or pass away, and this demographic event is not recorded in population registers, leading to population size overestimation. The errors introduced by population over-coverage on the assumed size, but also on the demographic composition and outcomes of a population, impact decisions made regarding the population, in particular its migrant component. Over-coverage has been reported to distort the denominators of national and migrant-specific rate estimates, especially when comparing migrants to native-born populations. Recently,^6^ found that correcting for over-coverage considerably affected mortality and fertility rates among migrants in Sweden. The magnitude of these errors suggests that over-coverage could undermine much of what is known about outcomes of migrant populations in host countries. In the worst-case scenario, these errors may misdirect research, misinform policy, and fuel harmful public narratives. MSE approaches have been considered for studying and correcting for over-coverage in Sweden^7^ using incomplete and overlapping population registers. These recent findings by^7^, highlighted the importance of employing modelling approaches, instead of deterministic ad-hoc rules, for estimating population size from register data. However, MSE approaches do not allow following of individuals across years, as they consider the register data of each year separately, and rely on modelling approaches for the corresponding contingency table data. Therefore, these approaches can only infer the population size in a given year, but cannot be used to infer patterns of emigration, either permanent or temporary.

In such cases, where individuals are followed over time, as is the case in the motivating data from Sweden, capture-recapture (CR) models^8^ provide a natural alternative to MSE approaches for studying populations and estimating corresponding demographic parameters, such as size, survival, and emigration/immigration patterns. In the case of CR models, individuals are caught in repeat sampling occasions and newly caught individuals are uniquely marked before being released back to the population, and can later be recaptured in subsequent sampling occasions. CR approaches are well-established for studying animal populations^9,10^, but, as mentioned above can also be relevant in the study of human populations when individuals are followed over time^11–13^. In the case of human populations, the sampling occasions correspond to different registers, where an individual can be recorded on all, any, or none of the registers in a given time period, such as a year, and register data for each individual are collected across time, such as in different years^14–17^.

In this paper, we build a CR model tailored to the Swedish register data, and showcase that CR models provide a methodological approach to estimate over-coverage in population registers, incorporating and exploiting the complexity of human international movements and behaviors. We consider migrants who arrived in Sweden between 2003 and 2015. All individuals register in the year they arrive in the country, and then can appear in a number of overlapping registers, such as the employment register or the internal moves register. All deaths in the country are recorded in the corresponding register, whereas individuals who leave the country can choose to de-register, and hence appear in the corresponding register. Individuals who have de-registered will re-register upon re-entry, if they return to the country, whereas for individuals who did not de-register it is unknown if and when they return. Based on this information, we model all probabilities related to migration movements and also related to appearing in the different registers when present in the country. Given these probabilities we are able to estimate over-coverage in Sweden from 2004 to 2016, considering a cohort CR-based approach with individuals who first entered the country between 2003 and 2015.

As more countries move to register-based systems for population data collection, the relevance of bias due to over-coverage is increasing. This issue has been found particularly problematic for migrant populations^18^, which now constitute one-fifth of Sweden’s residents^19^ and are more prone to administrative errors of this type due to higher mobility^20^. Our CR-based approach provides a general framework that can be tailored to different registers to provide reliable estimates of population size. Throughout the paper, we compare our method with a register-trace approach^6^, where individuals are considered to be in the country if they appear in at least one register in a given period. We also consider the model-based approach defined in^7^. This gives a necessary contrast between a fully deterministic method and probabilistic methods to estimate over-coverage using register data.

## Methods

### Data

In Sweden, individuals who plan to reside in the country for at least one year and have the legal right to do so must register with the Swedish Tax Authority. Registration is crucial for accessing public services and participating in society, as it provides a personal identification number required for various formal interactions, such as opening a bank account or obtaining a mobile phone. This mandatory registration ensures minimal under-coverage of the *de jure* population, except for those awaiting immigration status processing. Conversely, when emigrating, individuals must de-register if they plan to live outside Sweden for at least one year, though the incentives for de-registration are much lower compared to registration.

Our study population includes all foreign-born individuals who arrived in Sweden for the first time between 2003 and 2015 at age 18 or older, totaling 1,076,854 people. This group of immigrants is diverse, with substantial numbers from Iraq, Syria, Poland, Bosnia, and other parts of former Yugoslavia. The final year of observation is 2016, so every individual is part of the study population for at least one year after their arrival. From this population, a 5% random sample is taken, due to computational limitations of our method, as is explained in the next subsection. In short, we use one latent variable for each individual and each observation year, and these latent variables are sampled during the Markov chain Monte Carlo (MCMC) algorithm for all cases where the presence of an individual is unknown. Therefore using the whole population is currently impractical using this method.

Data from comprehensive administrative registers, including the Total Population Register, Longitudinal Integrated Database for Health Insurance and Labour Market Studies (LISA), the Intergenerational Register, and the Internal and International Moves Register, are used. These registers provide detailed information on various aspects of the individuals’ lives, such as demographic details, employment status, education, and family income, all compiled through personal identification numbers by Statistics Sweden. The complete list of registers and the indicators considered here in this paper are shown in the Supplementary Material S1.

Specifically, the study considers the information of presence in the registers, which is coded as 1 for yes and 0 otherwise. These registers include family income, indicating households with at least one member having positive annual income from various sources; enrollment in higher education during the autumn term; unemployment, indicating individuals actively searching for work; and employment, describing individuals employed or self-employed in November. Other registers include obtaining Swedish citizenship, birth of a child, transition to married or registered partnership, transition to divorced or separated partner, internal moves within Sweden, emigration, re-immigration, and death. These lists draw from sources such as the National Tax Authority, National Government Employee Pensions Board, National Social Insurance Authority, Swedish Public Employment Service, and the Educational Register.

### Model

We denote the number of individuals in the population, that is individuals registered in the country during the observation period, by *I*. The number of years, i.e. the observation period, is denoted by *Y* and the number of lists by *L*. Let $i = 1,\ldots ,I$ index individuals, $y = 1,\ldots ,Y$ years and $l=1,\ldots ,L$ lists. Each individual is registered when they first enter the country, with the registration time of individual *i* denoted by $r_i = \{1, \dots , Y\}$. The observation for individual *i*, list *l*, year *y* is denoted by $o_{iyl}$, where $o_{iyl} = 1$ if individual *i* was observed in list *l* in year *y*, and 0 otherwise.

For each individual we also have information about their sex, age, country of birth and time since first migration, measured in years. Previous studies have found that these are important factors for the probability of leaving the country^18^ and the probability of de-registering upon departure^6^. Countries of birth are grouped as 1) Denmark and Norway, 2) Iceland and Finland, 3) Eastern Europe, 4) Western, Europe, 5) Middle East and North Africa (MENA), 6) United States of America (USA), Canada and Oceania and 7) rest of the World.

We define a partially latent variable $Z_{iy} = \{0, 1\}$, which defines whether individual *i* was present in the country in year *y*. We assume that if individual *i* is seen in at least one list in year *y* then they are present that year, which can be defined as

$$\begin{aligned} \sum _l o_{iyl} > 0 \Rightarrow Z_{iy} = 1. \end{aligned}$$

We also define $Z_{ir_i} = 1$, i.e., all individuals are present in the year they registered.

We denote the survival indicator as $s_{iy}$, which is equal to 1 if the *i*th individual is alive in year *y* and 0 otherwise so that for individuals who died in year *y* $\{s_{iy-1} = 1, s_{iy} = 0\}$. We model the probability of surviving from year $y-1$ to year *y*, conditional on being alive in year $y-1$, for individual *i* as

1 $$\begin{aligned} \phi _{iy} = P(s_{iy} = 1 |s_{iy-1} = 1), \text{ where } \log \left( \frac{\phi _{iy}}{1-\phi _{iy}} \right) = {\varvec{x}}_{iy}^t {\varvec{\beta }}_\phi . \end{aligned}$$

We model the probability of staying in the country conditional on presence for each individual *i* and year *y*, as $\psi _{iy}$, and the probability of returning to the country conditional on absence, as $\eta _{iy}$. These probabilities are conditional on the state of the individual in year $y-1$, i.e. the probability of leaving in year *y* is conditional on being present in year $y-1$ and the probability of returning in year *y* is conditional on being absent in year $y-1$. We link these probabilities to covariates similarly to Eq. (1), with $\beta _\psi$ and $\beta _\eta$ as the parameters in the corresponding linear predictors. Based on these two probabilities and the survival indicator, we assume the following for the latent variable $Z_{iy}$

$$\begin{aligned} Z_{iy} | Z_{iy-1}, s_{iy} = 1 \sim \text{ Ber }\left( Z_{iy-1} \psi _{iy} + (1 - Z_{iy-1}) \eta _{iy}\right) . \end{aligned}$$

If we write $\theta _{iyl} = P(o_{iyl} = 1|Z_{iy}=1)$, then we model these probabilities as follows

$$\begin{aligned} \log \left( \frac{\theta _{iyl} }{1-\theta _{iyl}}\right) = {\varvec{x}}_{iy}^t {\varvec{\beta }}_o^{(l)}, \quad l=1,\ldots ,L, \end{aligned}$$

where ${\varvec{x}}_{iy}$ is a vector with the respective covariates sex, age, country of birth and time since first migration for individual *i* in year *y*, with the value 1 in the first position to estimate an intercept in the linear predictor.

We observe the death of individual *i* in year *y* ($d_{iy} = 1$) only if they are in the country that year ($Z_{iy} = 1$). Additionally, for each individual, we have their de-registering information, $e_{iy}$, which is equal to 1 if individual *i* left the country in year *y* and notified the official authorities, and 0 otherwise. We assume that if individual *i* leaves the country in year *y* they will de-register ($e_{iy} = 1$) with probability $\lambda _{iy}$,

$$\begin{aligned} \lambda _{iy} = P(e_{iy} = 1|Z_{iy} = 0, Z_{iy-1} = 1), \text{ where } \log \left( \frac{\lambda _{iy}}{1-\lambda _{iy}} \right) = {\varvec{x}}_{iy}^t {\varvec{\beta }}_\lambda , \end{aligned}$$

If we write ${\varvec{\beta }}_\Omega = ({\varvec{\beta }}_\theta ^{(l)}, {\varvec{\beta }}_\phi , {\varvec{\beta }}_\psi , {\varvec{\beta }}_\eta , {\varvec{\beta }}_\lambda ), \, l=1,...,L$, then our full model with our prior distribution for ${\varvec{\beta }}_\Omega$ can be defined as

2 $$\begin{aligned} {\varvec{\beta }}_\Omega&\sim N(0, \delta I) \end{aligned}$$

3 $$\begin{aligned} s_{iy} | s_{iy-1} = 1, {\varvec{\beta }}_\Omega&\sim \textrm{Ber} (\phi _{iy}) \end{aligned}$$

4 $$\begin{aligned} Z_{iy} | Z_{iy-1}, s_{iy} = 1, {\varvec{\beta }}_\Omega&\sim \textrm{Ber} \left( Z_{iy-1} \psi _{iy} + (1 - Z_{iy-1}) \eta _{iy}\right) \end{aligned}$$

5 $$\begin{aligned} e_{iy} | Z_{iy} = 0, Z_{iy-1} = 1, s_{iy} = 1, {\varvec{\beta }}_\Omega&\sim \textrm{Ber}(\lambda _{iy}) \end{aligned}$$

6 $$\begin{aligned} o_{iyl} | Z_{iy} = 1, s_{iy} = 1, {\varvec{\beta }}_\Omega&\sim \textrm{Ber}(\theta _{iyl}) \end{aligned}$$

We consider a Bayesian estimation process, where we use a normal distribution with mean 0 and variance equal to 2 as a prior distribution for every element of parameters ${\varvec{\beta }}_\phi , {\varvec{\beta }}_\eta , {\varvec{\beta }}_\psi , {\varvec{\beta }}_\lambda$ and ${\varvec{\beta }}_o^{(l)}$. We use NIMBLE^21^ to sample from the posterior distribution of all parameters and latent variables to ease the computational burden and employ their fast implementations for this type of models. This framework has been used in many applications in ecology^22,23^ and it allows for different algorithms in the estimation process^24,25^. We obtained 15,000 draws from the posterior distribution, discarded the first 5000 as burnin and kept every 10th value to decrease the autocorrelation within the chain. We retained two chains to check for convergence and Rubin-Gelman^26^ statistics were close to 1 for all parameters.

Over-coverage calculation takes into account estimates for latent variables $Z_{iy}$ considering a combination of posterior estimates when individuals are not seen in any list, $\hat{Z}_{iy} = E(Z_{iy}|\beta _{\Omega }, s_{iy}, e_{iy}, o_{iyl} )$, if $\sum _l o_{iyl} = 0$ and the assumption of presence when they are seen in at least one register, $\hat{Z}_{iy} = 1$, if $\sum _l o_{iyl} > 0.$ Based on these values, we can define our estimate for over-coverage for the whole population in a given year *y*, or any sub-group $D_y$,

$$\begin{aligned} OC_y = 1 - \frac{\sum _{i \in D_y} \hat{Z}_{iy}}{|D_y|}, \end{aligned}$$

where $|D_y|$ is the number of individuals registered in the country for (sub-)group $D_y$ in year *y* removing all individuals who died or emigrated.

## Results

**Figure 1:**
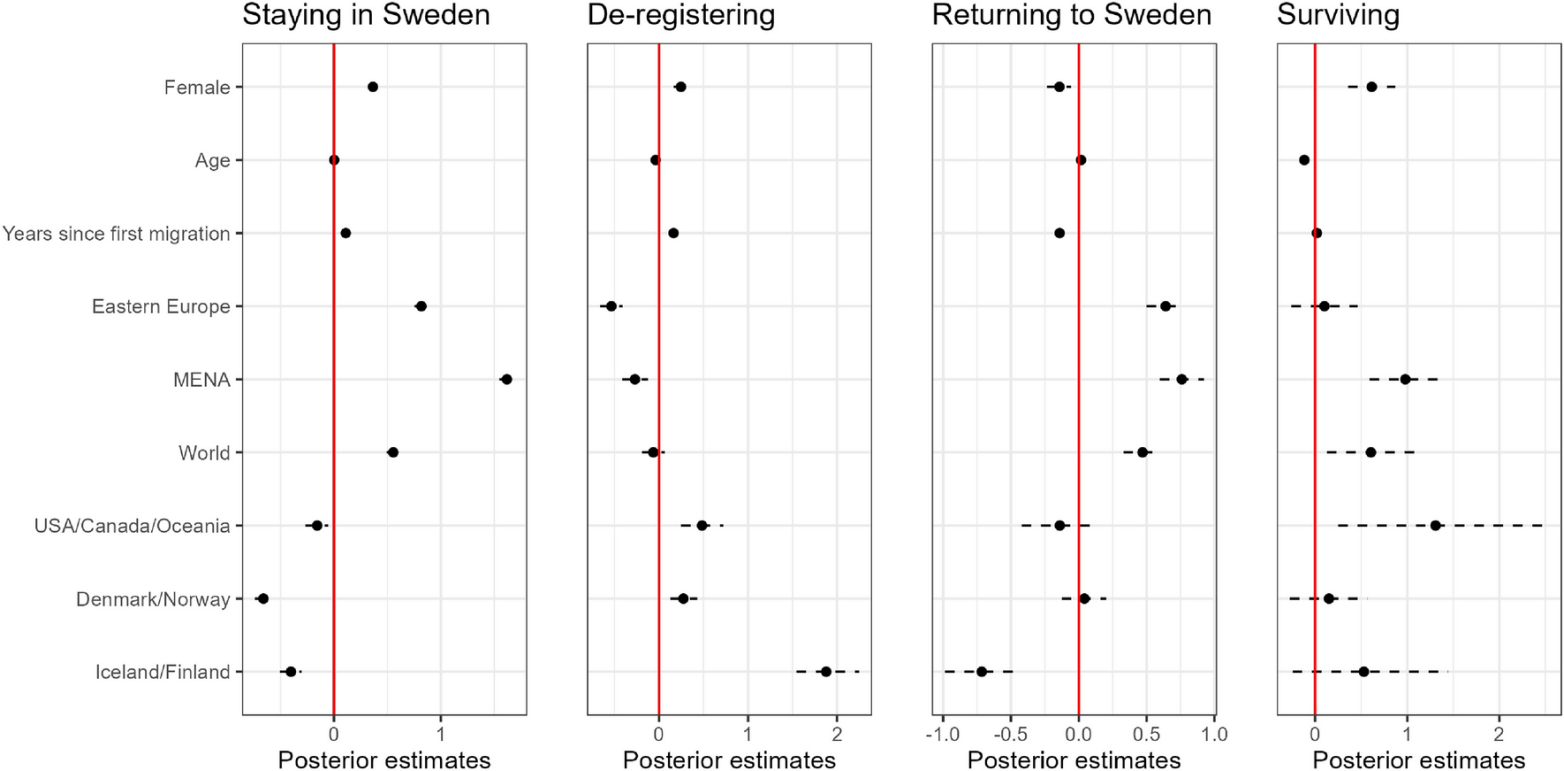
Posterior mean and 95% credible interval for each effect for the different linear predictors being considered in the model as discussed in the “Methods” section.

In Fig. 1 we summarise the posterior distributions of the effects of the different covariates in the distinct parts of the model for staying in Sweden, de-registering when leaving the country, returning to Sweden after emigration, and survival. The conclusions for each variable consider the levels of all other covariates fixed.

The probability of staying in the country ($\psi _{iy}$ in Eq. (4)), calculated via logistic model, is higher for females than males, does not change with age, increases with time since first migration, is higher for migrants from Eastern Europe and MENA (Middle East and North Africa), and is lower for people from the Nordic countries and USA/Oceania.

The part of the model which has the highest effect on over-coverage is related to the probability of de-registering when leaving the country, denoted by $\lambda _{ij}$ in Eq. (5), as in this case the primary source of over-coverage is individuals who emigrate without de-registering, as deaths in the country are registered with probability practically equal to 1, which is also our modelling assumption. We find that females have a higher probability of de-registering than males, and that the probability increases with time since first migration. Compared to migrants from Western Europe, migrants from Eastern Europe and MENA countries are less likely to de-register, while migrants from Nordic countries and USA/Canada/Oceania are more likely.

The probability for individuals to return to Sweden after having left the country, represented by $\eta _{iy}$ in Eq. (4), is higher for females than males, does not change considerably with age, decreases with the time since first migration, is higher for migrants from Eastern Europe and MENA, and is lower for people from Iceland/Finland.

**Figure 2:**
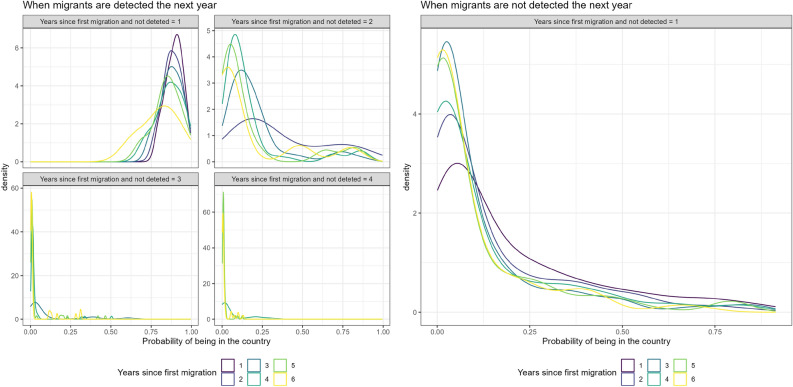
Density plot of the posterior probability of presence given the number of consecutive years that individuals have not appeared in any list, if (Left) they appear in at least one list the following year and (Right) if they do not appear in any lists the following year. Results are presented separately by time since first migration.

**Figure 3:**
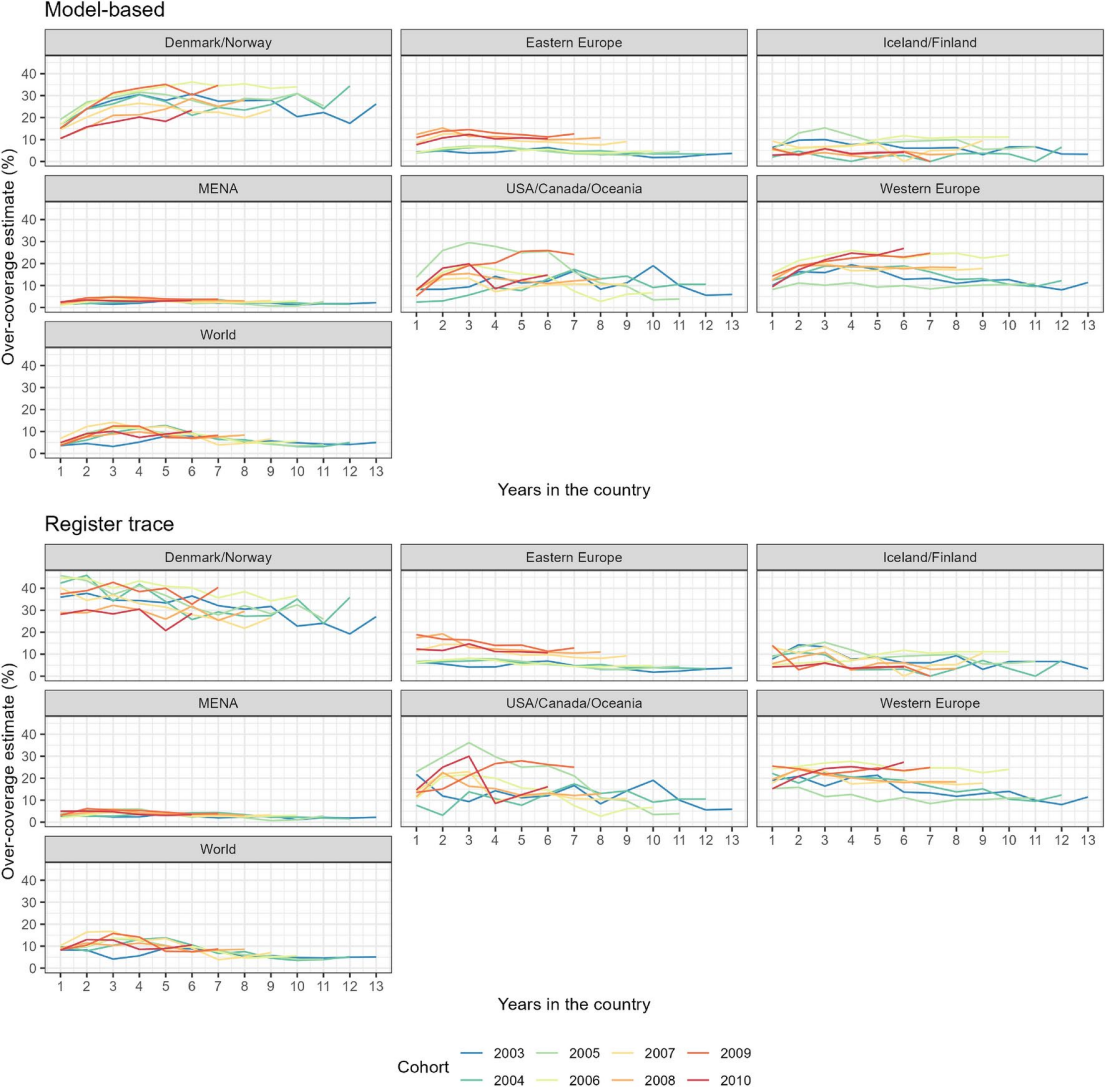
Over-coverage estimates by group of countries considering the model based approach (top panel) and the register trace approach (bottom panel), with different colors for each cohort.

Our model provides posterior summaries for the probability of being in the country for all individuals, conditional on their data, after their first migration. Figure 2 shows density plots of the posterior probability of presence given the number of consecutive years that individuals have not appeared in any list, if they appear in at least one list the following year and if they do not, with results presented separately by time since first migration. The numbers of individuals considered in each density line are listed in the Supplementary Material S1. First, when migrants are not detected for 3 or 4 consecutive years, then they have a very low probability of being in the country, regardless of their time since first migration. Furthermore, as time since first migration increases, the probability of being in the country for individuals not detected decreases, regardless of the number of years since their last detection. When migrants are not seen for only one year, their probability of being in the country given no detection is quite high, and specifically mostly greater than 0.5 for individuals who first migrated to Sweden six or more years before the year considered, and greater than 0.75 for individuals who first migrated to Sweden one year before the year considered. When individuals are not seen for two consecutive years (top right corner on the left side of Fig. 2), then the distribution of the probability of being in the country is concentrated at values lower than 0.25. A similar pattern is observed on the right side of Fig. 2, when migrants are not detected in any list in the following year, as the corresponding distribution is concentrated at values lower than 0.5. For this scenario, we do not show the densities for cases when individuals are not detected for more than one year, since the corresponding probabilities are very close to 0.

All of these migrants, for which we can calculate their probability of being in the country shown in Fig. 2, would be labeled as over-covered in the year they are not detected by the register-trace approach^6^, as they are officially registered as part of the population but are not present in any of the registers available that year. Our longitudinal approach on the other hand borrows information across years and hence allocates a corresponding probability of presence for these individuals, which can be high when they are not seen for only one year, but then are detected in at least one list in the next year, for instance. For other cases, our model provides probabilities with a higher concentration around zero.

Finally, we summarise the probabilities of being detected in at least one list, for males and females according to their country of origin and time since first migration (Fig. 8 of the Supplementary Material S1) and the corresponding probabilities for all considered lists separately. The results suggest a lower probability for new arrivals, with the probability approaching one as time since first migration increases, and a lower detection probability for females compared to males, overall, and especially when considering the employment register.

**Figure 4:**
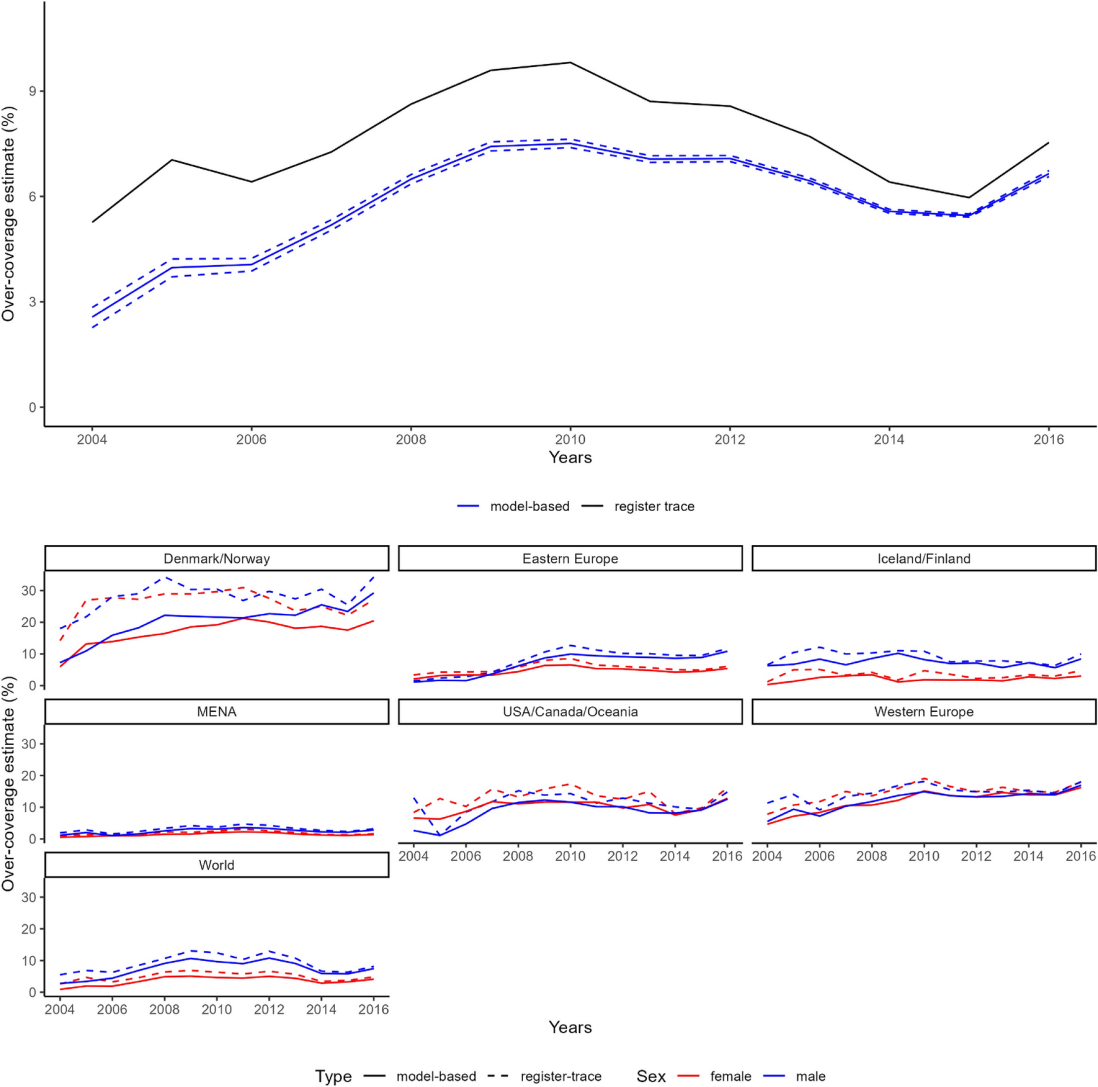
Top plot: Estimates of over-coverage over the years considering the register trace approach (black solid line) and considering our model-based approach (blue solid line, with 95% CI in blue dashed lines). Bottom plot: over-coverage model estimates for all combinations of sex and groups of country of birth.

In the next step of our analyses we compare all the different cohorts in the study by their time since their first migration, comparing our model-based estimates with those from the register-trace approach (Fig. 3). First, we notice how the register trace approach has a high estimate of over-coverage for newly arrived individuals. The estimates based on our model show a smoother increase for all cohorts and groups of countries. The results highlight that our model-based estimates for over-coverage can correct for non-detection in the different registers in the first years of stay, when migrants are still establishing themselves in their new host country.

In Fig. 4 we show our estimates of over-coverage for the total study population consisting of all migration cohorts arrived between 2003 and 2015 considered in this study. There is a bigger difference between over-coverage estimates in the early years of the study because the study population at that point only consists of newly arrived individuals. The register-trace approach only considers the individuals who are known to be present, since they appeared in at least one list, whereas our approach also allows for individuals to be present but not detected, hence in the years shortly after arrival to the country, when individuals are less likely to be observed, the difference between the two approaches is more marked. As more cohorts are added to the study, the difference between the two methods lessens. In the bottom plot, we have over-coverage estimates for all combinations of group of countries and sex. Specifically, we can see that this effect of a smaller difference throughout the years varies for the different groups of countries. For instance, for Denmark and Norway, for females there is a big difference between the register trace approach and the model-based for all the years of the study. For Eastern Europe, there is a big difference between the two methods around 2010, but for males year 2004, the first year of over-coverage estimation, the difference is small.

**Figure 5:**
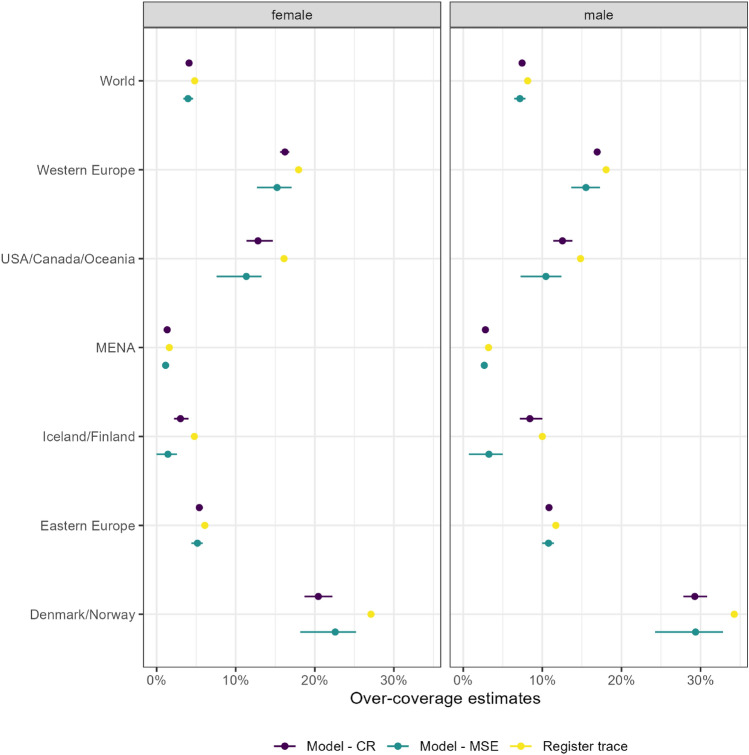
Comparison of over-coverage estimates and their respective 95% credible interval in year 2016 based on the register-trace approach (yellow), the MSE model (green) and the new approach, using a capture-recapture (CR) model (purple), for female (left) and male (right) migrants. The register-trace approach is a deterministic method, so it does not have a credible attached to its estimate of over-coverage, while the other two methods might present narrow intervals.

A previous attempt in modelling the uncertainty regarding the presence of migrants considered MSE models, which rely on cross-sectional views of the migrant population on yearly basis. Our approach differs from this first attempt as we follow each migrant trajectory and we use their whole history in the estimation process. Hence the comparison between the two approaches is difficult, but here we show the results for the two methods using the information from year 2016 in Fig. 5. Except for Denmark/Norway, our proposal shows slightly higher estimates of over-coverage compared to the estimates based on MSE. This might be explained by the accumulation of data until 2016, which mitigates the uncertainty and the difference to the register approach is explained mainly by nearly arrived migrants, as they have a higher probability of being in the country, as discussed previously. As the MSE approach only uses information for one year alone, it estimates more people being in the country, when these migrants are not detected in any list, as it is unable to borrow information from previous years. Additionally, our approach provides narrower credible intervals, as it is able to use more information efficiently on the estimation of over-coverage. Furthermore, both probabilistic approaches have estimates of over-coverage smaller than the register-trace approach.

Overall, the estimates based on our approach are to some extent similar to those provided using the MSE. Besides providing narrower credible intervals, we are able to directly calculate probabilities of staying in Sweden, de-registering when they leave the country, returning to Sweden when they have left and surviving throughout the years. Examples of all these probabilities are shown in the Supplementary Material S1. Therefore our method is able to provide reasonable probabilities of being in the country, whether high values when migrants are not seen for one year only or values close to zero when they are not seen for many years, while also explaining different aspects of migration movements, such as emigration and/or re-immigration, with probabilities attached to each component.

### Examples of profile probabilities

We consider some demographic profiles in our random sample, with the aim of highlighting one of the main contributions of our approach, which is the possibility of estimating the probability of presence for each individual and year, given their personal characteristics, sex, age, time since first migration and their country of birth. These profiles were described in a way that represents at least 10 individuals in the population (among the random sample). We do not provide further details for privacy reasons, but the model estimates individuals probabilities.

The first example is a female, who migrated to Sweden in 2005, and was detected in at least one list in the last year of the observation period (2016). This migrant from Eastern Europe was detected in the family income list almost every year, with the exception of three years in the study period. Only in 2008 she was not seen in any list. The register trace approach would consider this woman over-covered in this particular year, i.e., not present in the country. Our model gives a posterior probability of 0.8275 of her being in the country that year.

The second example is a male from a MENA country, who entered the country in 2006 and was also detected in at least one list in the last year of the observation period (2016). This migrant was detected in the family income list almost every year, with the only exception of 2009. He was also present in the employment list in many years throughout the study period and in three different years in the internal moves list. In 2009, when he was not present in any list, the register-trace approach would deem this individual over-covered, while our model gives a posterior probability of 0.839 of him being in the country that year.

The last example considers a case where the migrant was not seen in any list for two straight years. This woman from Western Europe arrived in 2007 and had a child in 2008. She was not detected in any list for two years after having a child. She appeared after that in the internal moves list and also in the family income. The posterior probabilities of presence in the country are 0.429 and 0.428 in the first and second year not seen, respectively, so as expected appreciably lower than in the above cases where individuals were only missed for one year.

## Discussion

The previous literature has explored estimating over-coverage in Sweden by employing methods such as multiple system estimation (MSE) models^7^ or a deterministic register-trace approach^6^. These approaches, however, analyze data cross-sectionally, assessing each year independently (or adding information for the year before and after) without the ability to integrate information across different years and overlooking the dynamics of temporary emigration. In contrast, our study adopts a longitudinal perspective, tracking migrants during their initial years in Sweden - a period when they are known to be at a higher risk of over-coverage^7^. By following a capture-recapture model with recovery and temporary emigration, we monitor individuals from their arrival, modeling their annual probabilities of survival, emigration, and the probability of deregistering and returning if they depart.

Our proposal using capture-recapture models provides a scheme which learns from all events related to migrating populations, such as leaving and/or returning to the country, de-registering when leaving, engaging in socioeconomic activities in their new country, attaching probabilities to all these matters and sharing this knowledge amongst all individuals in the sample. This allows for a more complete picture of the migrant trajectory in their new country and consequently it enables a more accurate estimate of over-coverage in Sweden. Our approach enhances demographic research by applying a lifecourse perspective and enabling the integration of population processes. Demographic research, particularly in the context of migration, addresses highly complex and interdependent processes. This complexity necessitates a modelling strategy that is both sophisticated and straightforward. By using capture-recapture models to estimate over-coverage, we not only improve the precision and depth of demographic studies but also ensure a more comprehensive understanding of migrant trajectories.

We have used a 5% sample in our endeavor due to computation limitations, as larger values of the population were unfeasible to be used, as each additional individual requires a latent variable to be estimated for each year they are not detected after their arrival in Sweden. As a consequence, computation time increases drastically and the requirement of a larger memory for these computations make this task impractical. In this sense, the development of models for large data sets^27,28^ are essential for further understanding of the dynamics of over-coverage for migrant populations. Nevertheless, our method still uses a sample large enough to provide fair estimates for the posterior means of the parameters, as we consider more than 35,000 individuals in this effort. In the supplementary material S1, we have added a table with the values of arrivals of migrants by year and groups of countries and a plot to show its respective distribution over the years.

This paper contributes to the literature also providing an individual estimation of presence in the territory that has the potential to be used in future studies to adjust the denominator when calculating rates or as weight in regression models.

## Supplementary Information

Supplementary Information.

## Data Availability

This study is produced under the Swedish Statistics Act, where privacy concerns restrict the availability of register data for research. Aggregated data can be made available by writing to Eleonora Mussino (eleonora.mussino@sociology.su.se), conditional on ethical vetting.
