# Supplementary material for: Using population register data and capture-recapture models to estimate over-coverage in Sweden
Source: Sci Rep. 2024 Dec 18;14:30551. doi: 10.1038/s41598-024-82547-9 (PMC11655540; doi:10.1038/s41598-024-82547-9)
Supplement: Supplementary file 1 — Supplementary Information. [file 41598_2024_82547_MOESM1_ESM.pdf]

Supplementary material for "Using register data and capture-recapture models to estimate over-coverage in Sweden"

Bruno Santos[1,*], **Eleonora Mussino**[2], **Sven Drefahl**[2], **and Eleni Matechou**[3]

[1]CEAUL – Centro de Estatística e Aplicações, Faculdade de Ciências, Universidade de Lisboa, Lisbon, Portugal
[2]Stockholm University Demography Unit -SUDA, Stockholm University, Stockholm, Sweden
[3]University of Kent, School of Mathematics Statistics and Actuarial Sciences, Canterbury, United Kingdom
[*]eleonora.mussino@sociology.su.se

ABSTRACT

Registers available

We list here all the administrative registers used in this paper and the indicators obtained from the respective register.

**Administrative register**	**Indicator**
Intergenerational register	Child born
Total Population Register [RTB]	Obtaining Swedish citizenship<br>Marriage<br>Divorce
Internal moves register	Internal move
Longitudinal Integrated Database for<br>Health Insurance and Labour Market Studies (LISA)	Family income<br>Employment<br>Unemployment<br>Higher Education

Additional information

In this supplementary material, we have added further plots to help understanding the results based on our model-based approach. First, we show in Figure 1 the estimates of over-coverage based in our approach and the register-trace approach for every combination of group of countries and sex. Following, in Figure 2, we add the model coefficients related to the probabilities of being observed in the different lists. In Figure 3 we show the distribution of groups of countries of migrants by year of their arrival, with the actual numbers shown in Table 1. Moreover, the absolute numbers of individuals considered in Figure 2 of the main manuscript for each density line is presented on Tables 2 and 3.

Summaries of probabilities

Here we provide some plots to illustrate the probabilities provided by the model as a function of the available variables. We consider three different values for age, 18, 35 and 60 years old and vary years

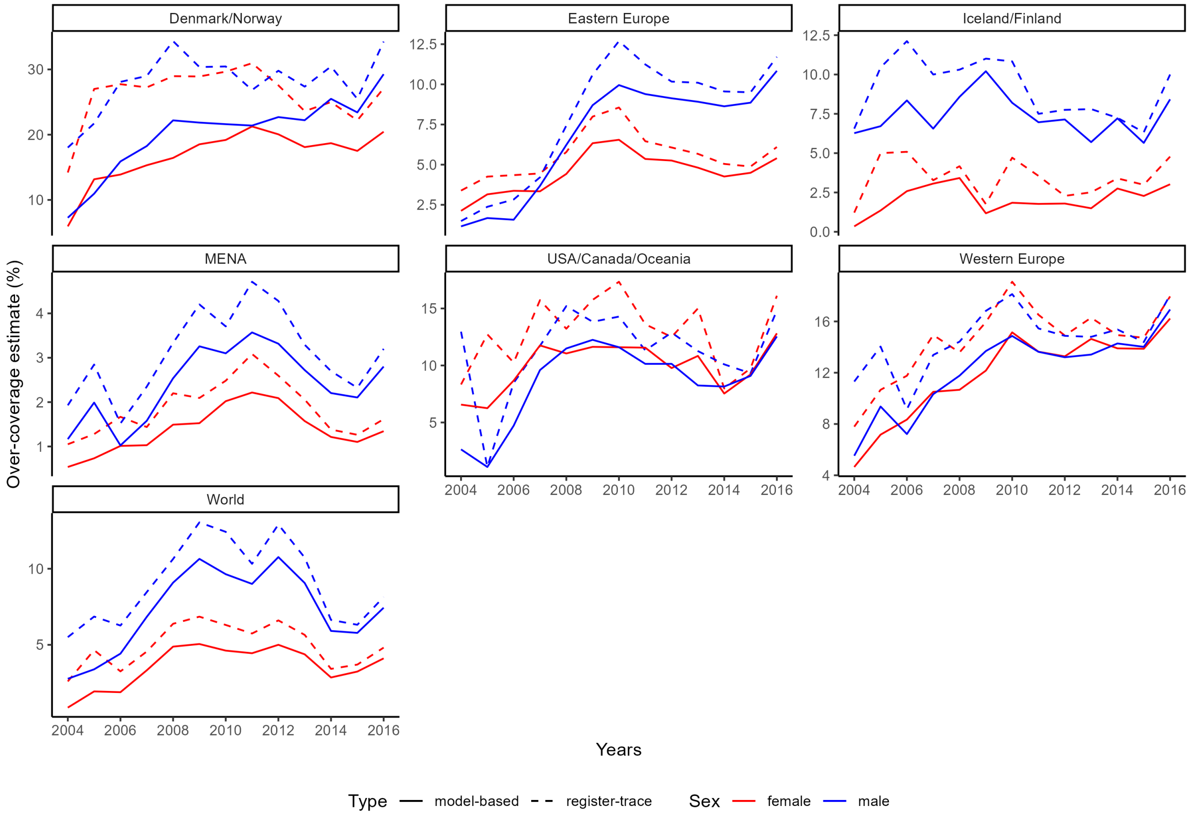

Figure 1. Over-coverage estimates for every group of countries considering the model based approach (solid lines) and the register trace approach (dashed lines), for males (blue lines) and females (red lines).

since first migration between 1 and 10 years. We also consider all groups of countries and both sexes when displaying these probabilities. It is important to note that each set of probabilities have a different scale, therefore all the different plots will have a different y axis. Although this setup makes the comparison harder, it does help to visualize the variation of these different probabilities.

	Den./Nor.	East. Europe	Icel./Finl.	MENA	USA/Can./Oceania	West. Europe	World	Total
2003	181	266	93	438	53	214	371	1616
2004	188	392	79	312	44	181	427	1623
2005	189	438	63	346	65	234	468	1803
2006	219	682	58	893	71	307	618	2848
2007	224	755	63	906	58	363	640	3009
2008	163	741	66	775	80	384	723	2932
2009	146	673	67	866	66	327	826	2971
2010	119	646	68	847	80	281	858	2899
2011	121	627	67	739	91	319	710	2674
2012	115	655	58	844	87	354	736	2849
2013	136	646	66	1043	84	344	788	3107
2014	89	707	78	1454	82	349	879	3638
2015	89	731	99	1543	74	357	963	3856

Table 1. Number of new arrivals of migrants by year and group of country in the sample considered for this study.

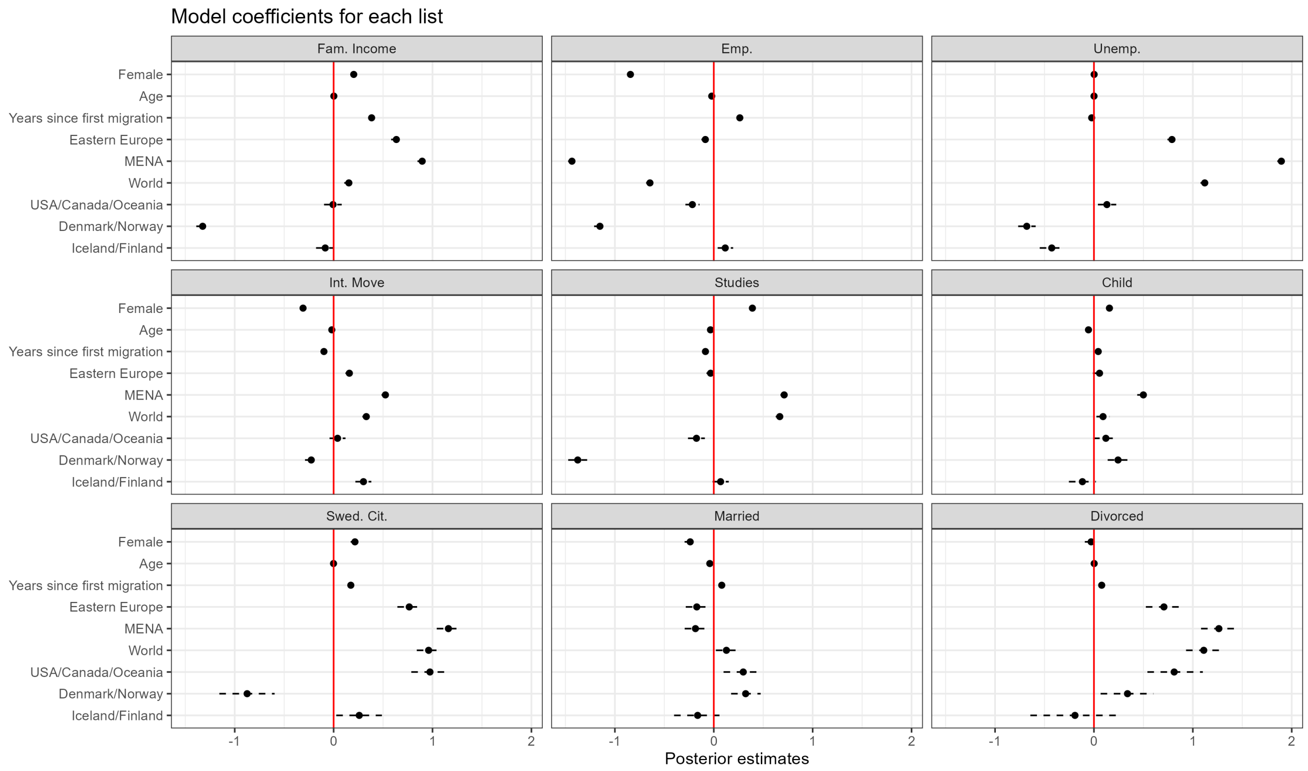

Figure 2. Posterior mean and 95% credible interval for each effect for the linear predictor considering the different lists in the model as discussed in the Methods section.

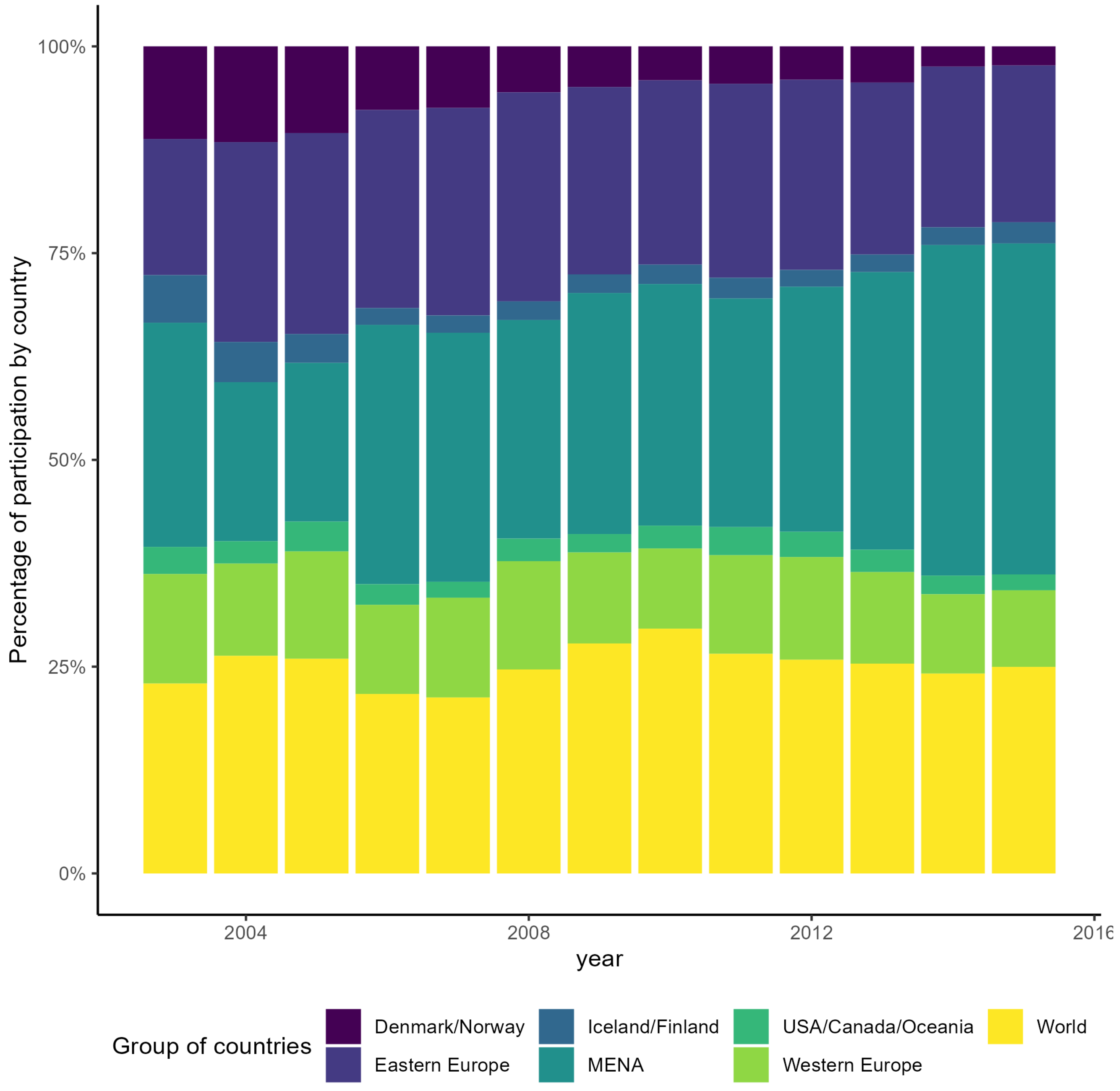

Figure 3. Distribution of groups of countries for new migrant arrivals by year.

	Years in the country					
Years not detected	1	2	3	4	5	6
1	1031	457	311	191	126	89
2	0	443	199	130	75	46
3	0	0	252	88	49	33
4	0	0	0	140	50	36

Table 2. Absolute numbers of migrants considered in the left side of Figure 2 in the main manuscript for each density line.

	Years in the country					
Years not detected	1	2	3	4	5	6
1	1837	794	463	300	189	153
2	0	1211	476	280	175	112
3	0	0	872	323	192	119
4	0	0	0	648	226	130

Table 3. Absolute numbers of migrants considered in the right side of Figure 2 in the main manuscript for each density line. For this plot, only when years not detected is equal to 1 is shown in the final version of this figure.

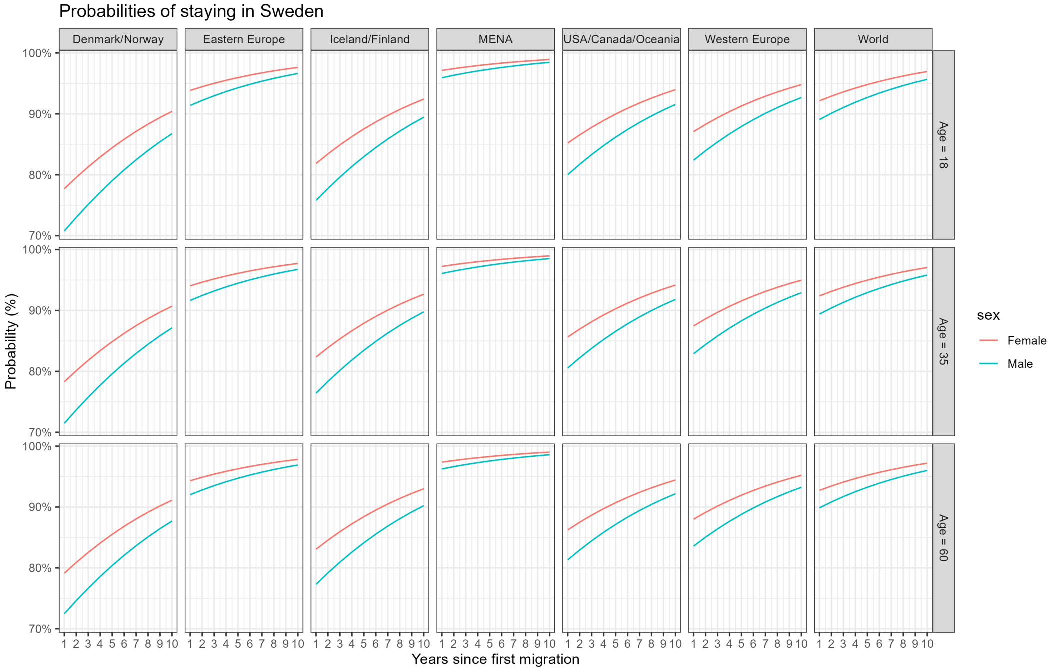

Figure 4. Estimated probabilities of staying in Sweden as a function of sex, group of countries, age and years since first migration.

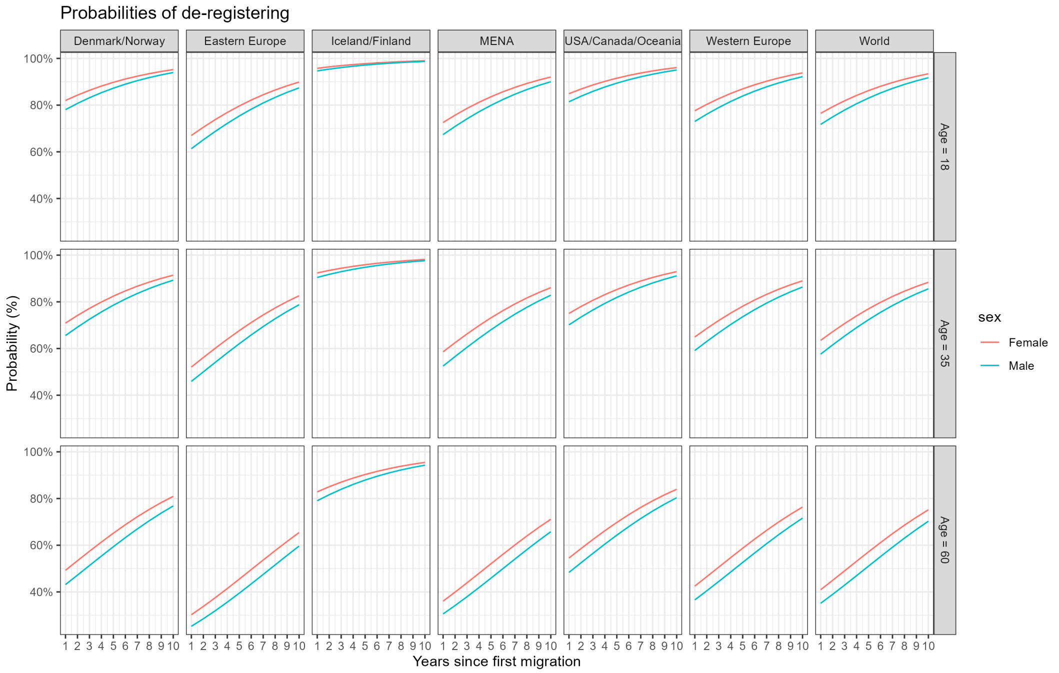

Figure 5. Estimated probabilities of de-registering as a function of sex, group of countries, age and years since first migration.

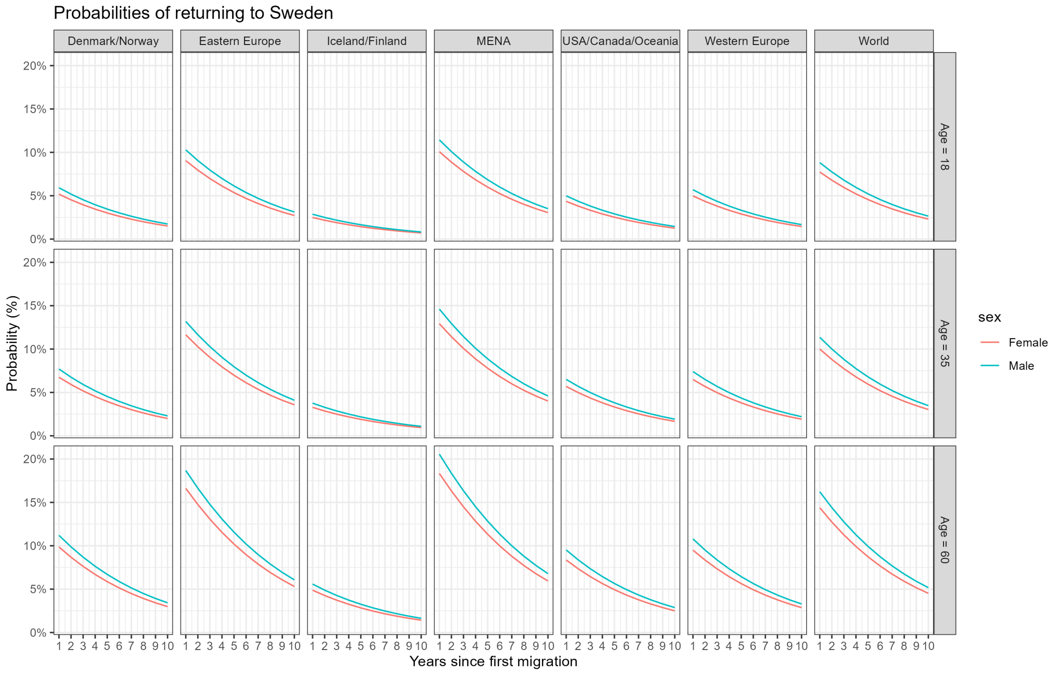

Figure 6. Estimated probabilities of returning to Sweden as a function of sex, group of countries, age and years since first migration.

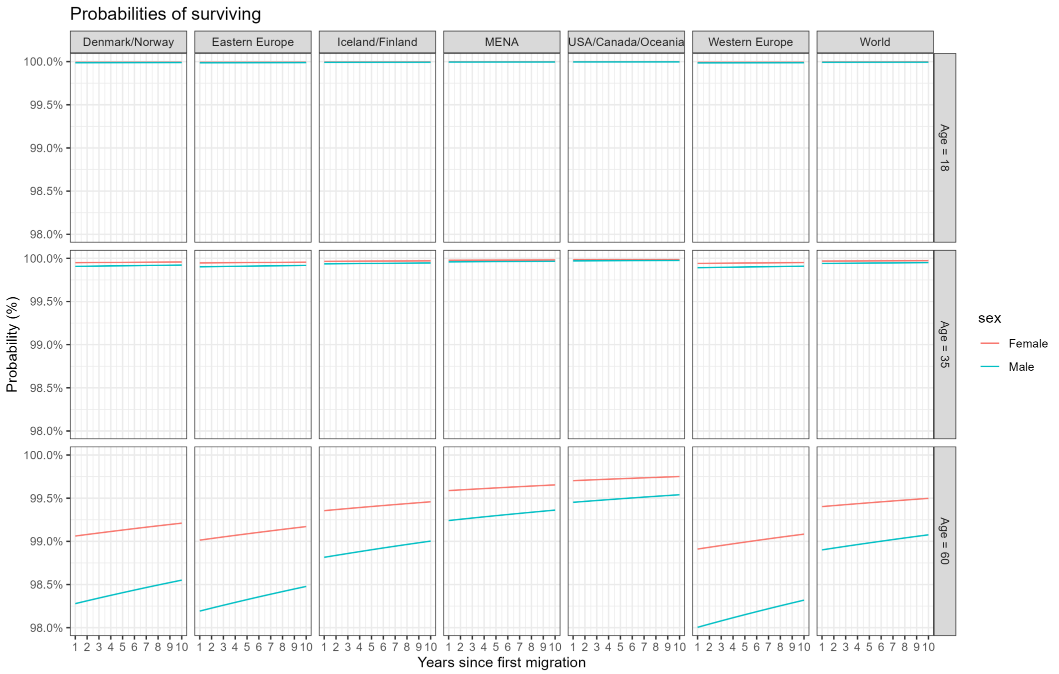

Figure 7. Estimated probabilities of surviving as a function of sex, group of countries, age and years since first migration.

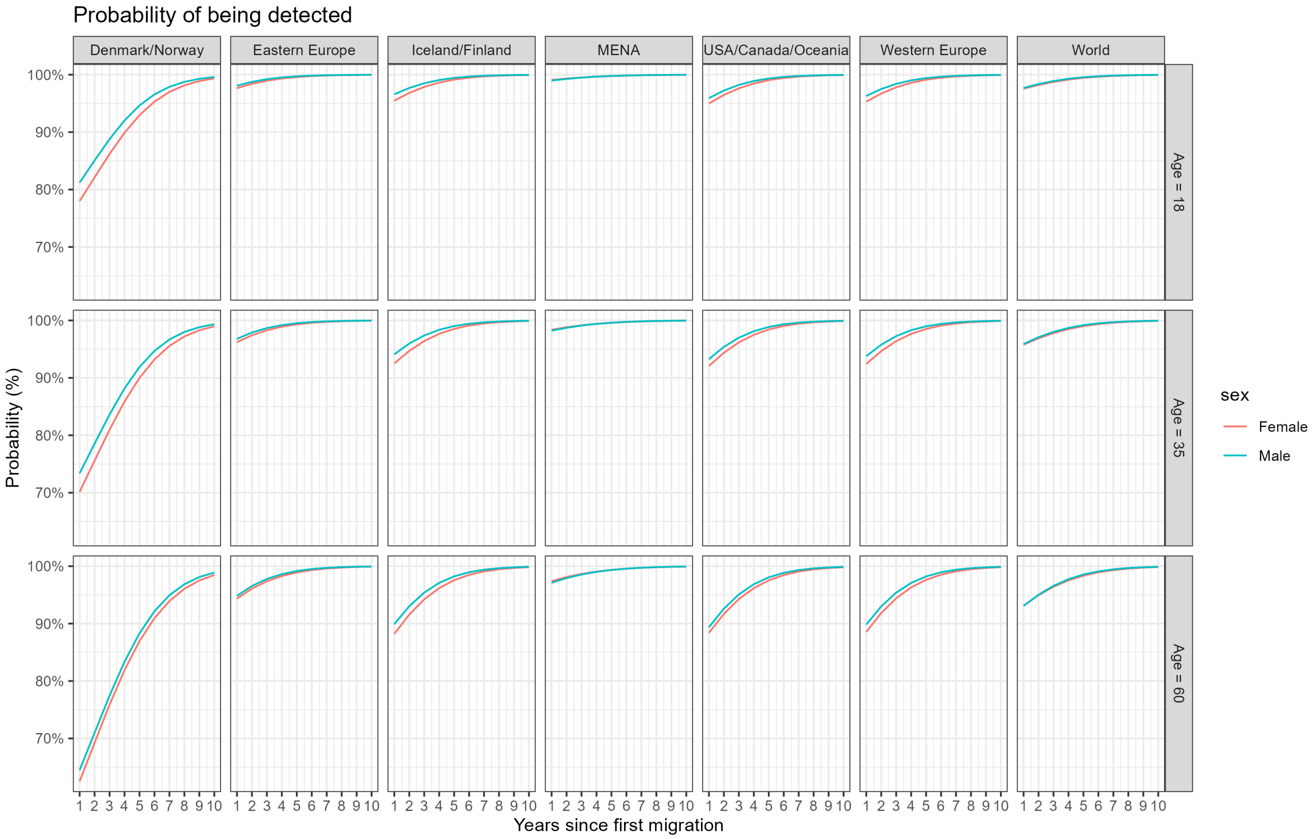

Figure 8. Estimated probabilities of being detected as a function of sex, group of countries, age and years since first migration.

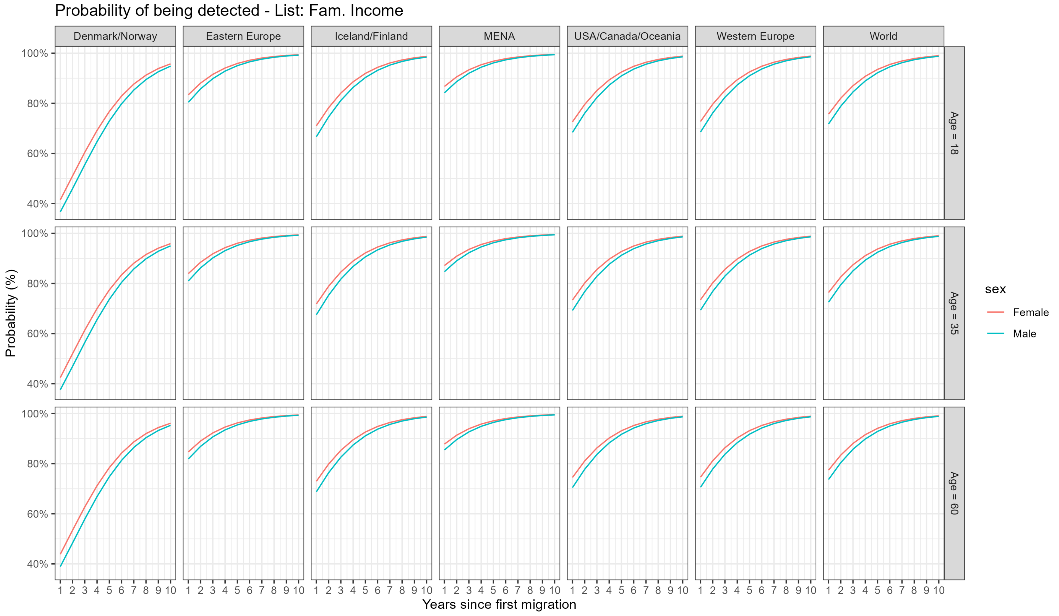

Figure 9. Estimated probabilities of being detected in the list of family income as a function of sex, group of countries, age and years since first migration.

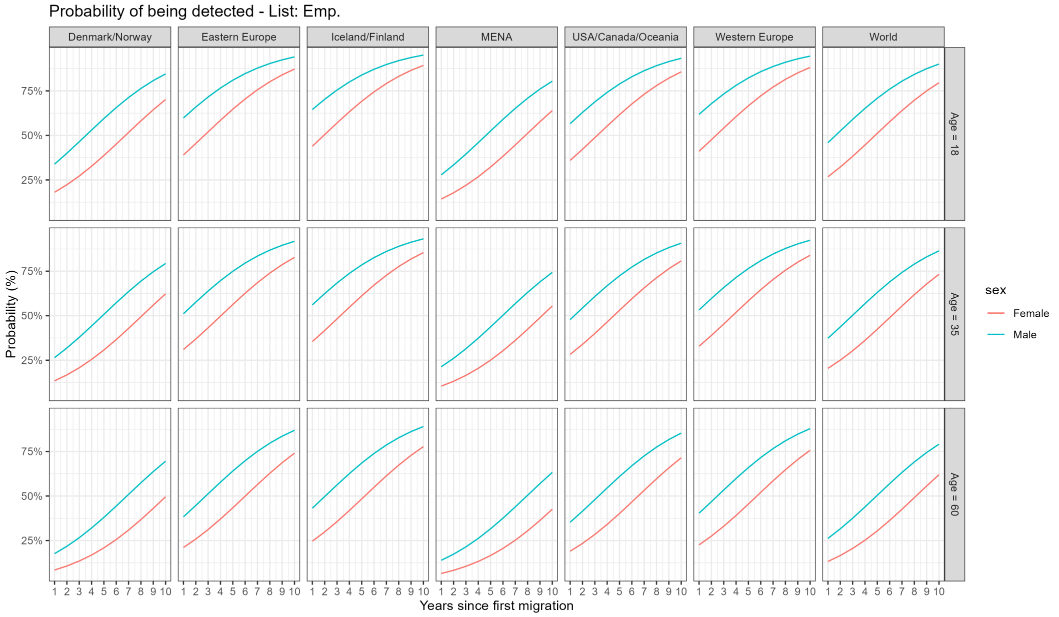

Figure 10. Estimated probabilities of being detected in the list of employment as a function of sex, group of countries, age and years since first migration.

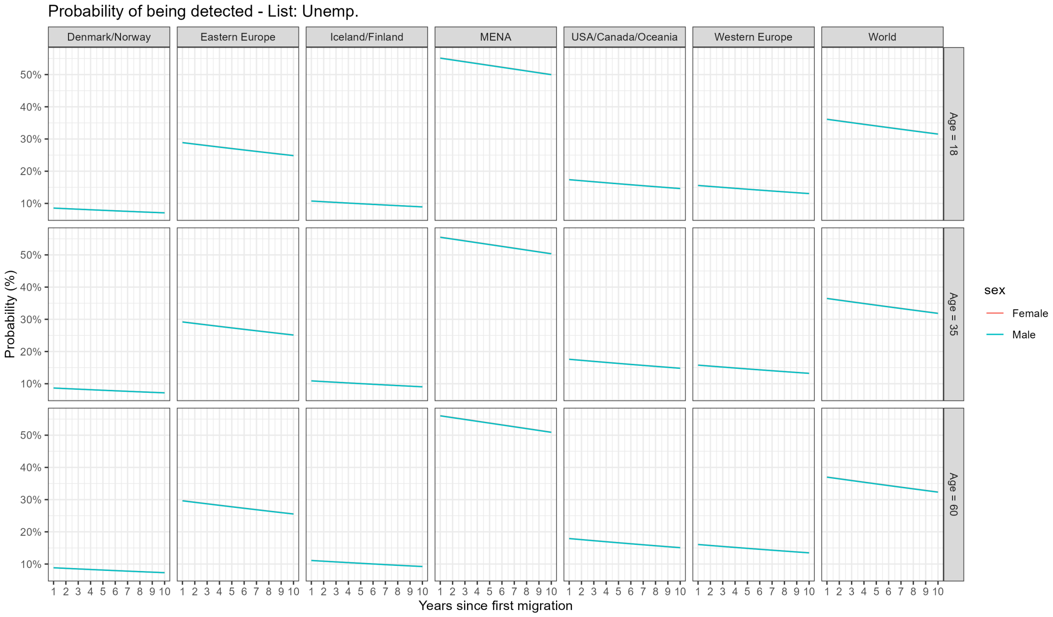

Figure 11. Estimated probabilities of being detected in the list of unemployment as a function of sex, group of countries, age and years since first migration. For this list, as the effect of sex is close to zero (Fig. 2), the blue line is plotted over the red line, so the red line is hidden.

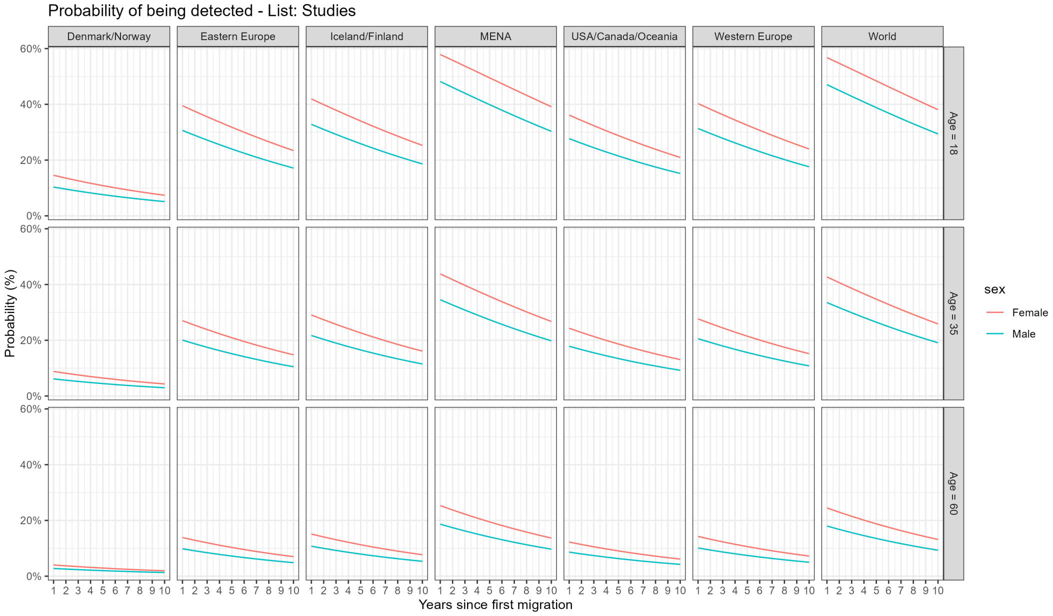

Figure 12. Estimated probabilities of being detected in the list of studies as a function of sex, group of countries, age and years since first migration.

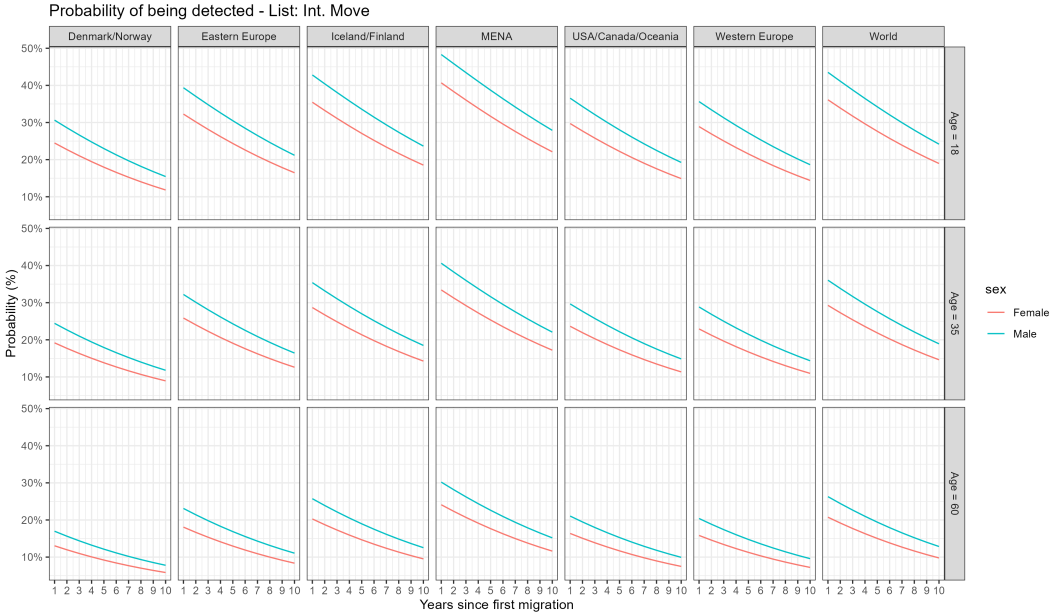

Figure 13. Estimated probabilities of being detected in the list of internal moves as a function of sex, group of countries, age and years since first migration.

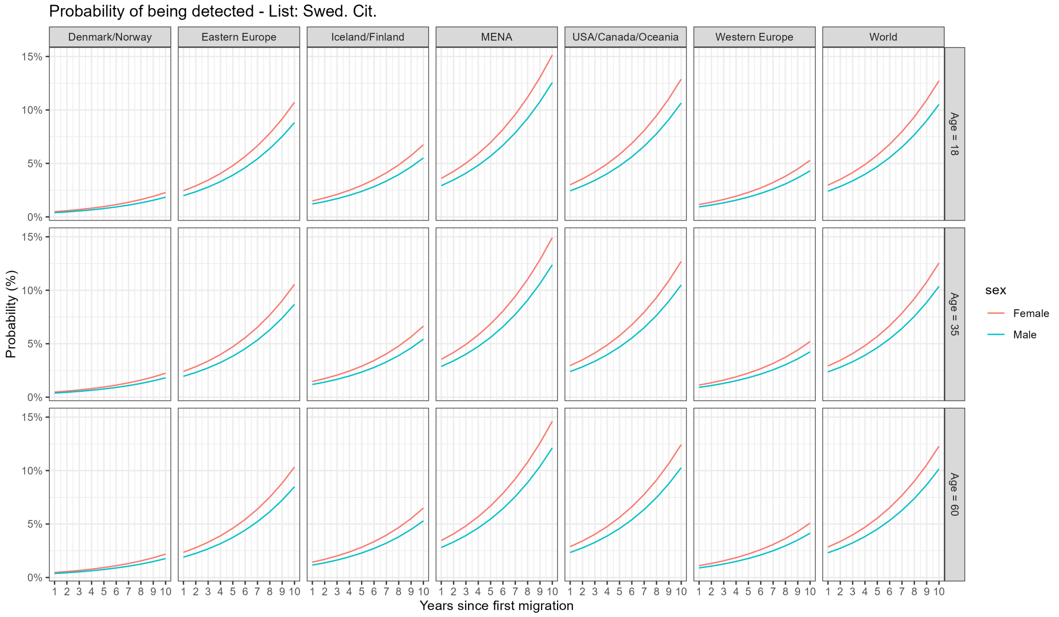

Figure 14. Estimated probabilities of being detected in the list of Swedish citizenship as a function of sex, group of countries, age and years since first migration.

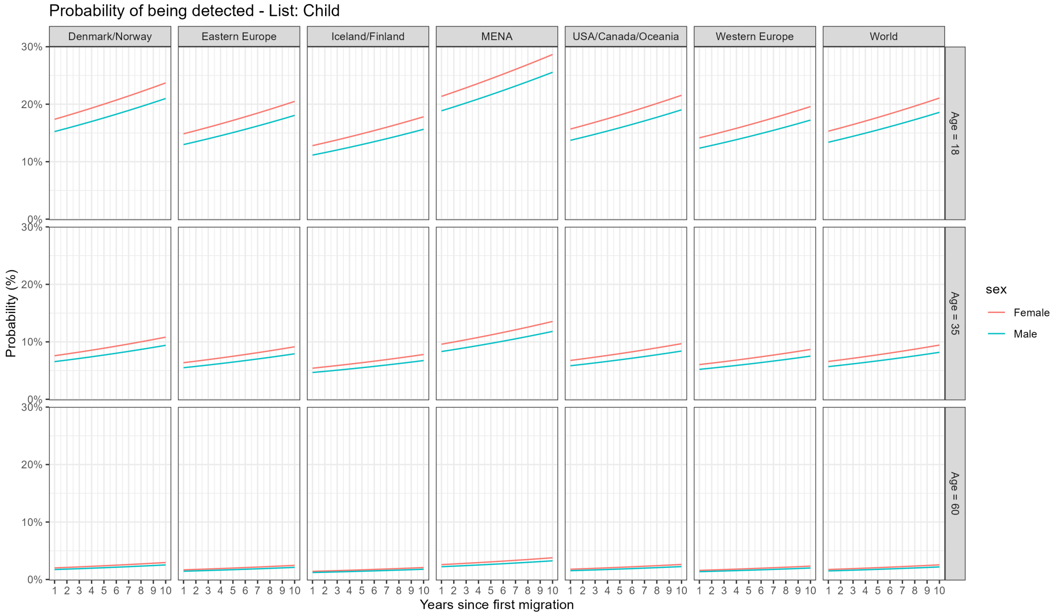

Figure 15. Estimated probabilities of being detected in the list of having children as a function of sex, group of countries, age and years since first migration.

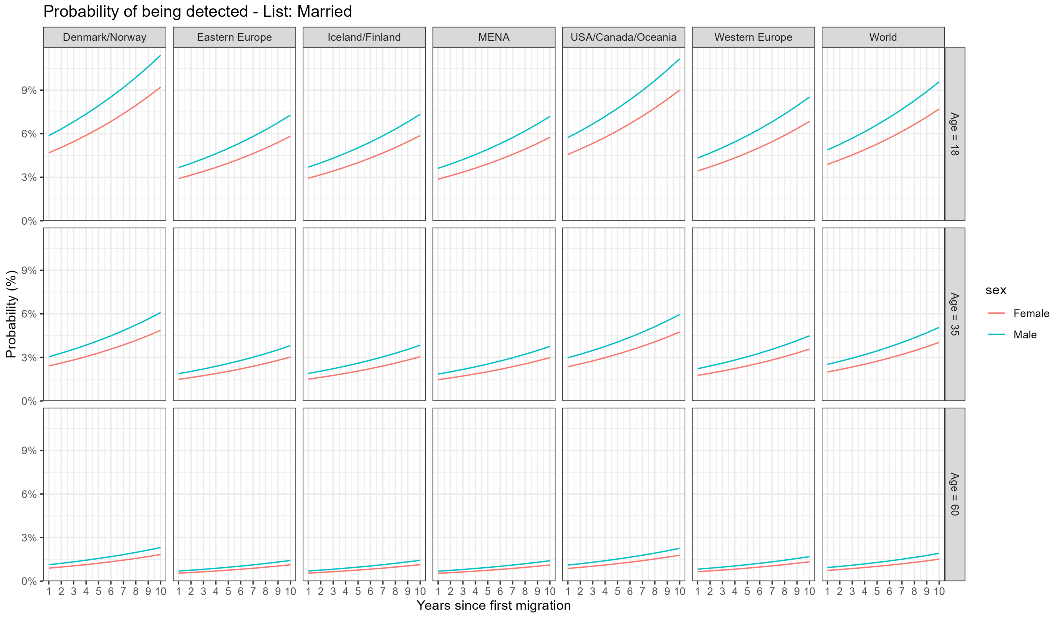

Figure 16. Estimated probabilities of being detected in the list of marriage as a function of sex, group of countries, age and years since first migration.

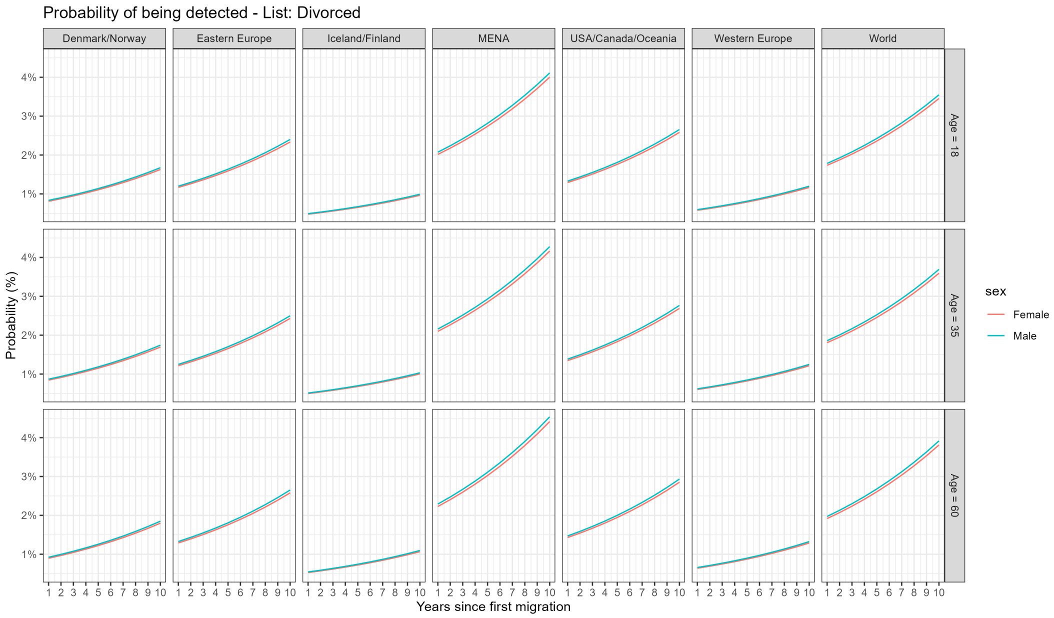

Figure 17. Estimated probabilities of being detected in the list of divorce as a function of sex, group of countries, age and years since first migration.
